# Modeling of Beams’ Multiple-Contact Mode with an Application in the Design of a High-g Threshold Microaccelerometer

**DOI:** 10.3390/s110505215

**Published:** 2011-05-11

**Authors:** Kai Li, Wenyuan Chen, Weiping Zhang

**Affiliations:** Key Laboratory for Thin Film and Microfabrication of Ministry of Education, National Key Laboratory of Nano/Micro Fabrication Technology, Research Institute of Micro/Nanometer Science and Technology, Shanghai Jiao Tong University, Shanghai, 200240, China; E-Mail: iamkaili@gmail.com

**Keywords:** multiple-contact mode, beam, high-g, threshold microaccelerometer

## Abstract

Beam’s multiple-contact mode, characterized by multiple and discrete contact regions, non-uniform stoppers’ heights, irregular contact sequence, seesaw-like effect, indirect interaction between different stoppers, and complex coupling relationship between loads and deformation is studied. A novel analysis method and a novel high speed calculation model are developed for multiple-contact mode under mechanical load and electrostatic load, without limitations on stopper height and distribution, providing the beam has stepped or curved shape. Accurate values of deflection, contact load, contact region and so on are obtained directly, with a subsequent validation by CoventorWare. A new concept design of high-g threshold microaccelerometer based on multiple-contact mode is presented, featuring multiple acceleration thresholds of one sensitive component and consequently small sensor size.

## 1. Introduction

Beam’s multiple-contact mode has been used in micromachined RF switches [1,2]. In this type of switch, there is an array of discretely distributed stoppers and an electrostatically driven cantilever, and stoppers contact the cantilever one by one in a zipper-like way. In fact, beam’s multiple-contact mode has a wider application perspective, for example it can be used in threshold microaccelerometers, microgrippers and so on. In this article, with a combination of the design of a high-g threshold microaccelerometer, a universal method and model will be developed for beam’s multiple-contact analysis.

**Figure 1.**
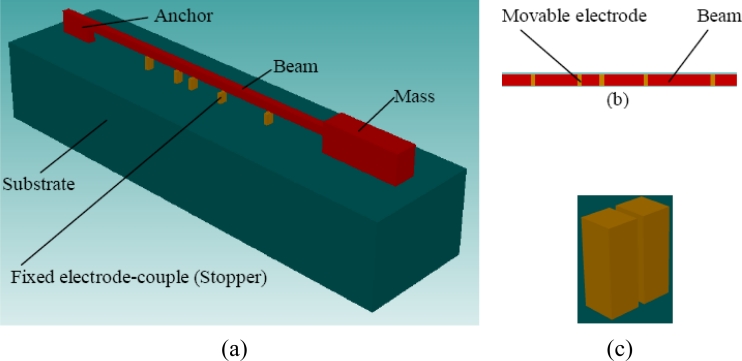
**(a)** Multiple-contact threshold microaccelerometer; **(b)** Movable electrode; **(c)** Fixed electrode-couple (Stopper).

The measurement of high-g acceleration has been widely studied [3–10]. Threshold microaccelerometers are one type of high-g microaccelerometer that has developed rapidly in recent years. Traditionally a threshold microaccelerometer is composed of an array of sensitive components which are usually beams or beam-mass structures of different sizes, and each sensitive component is only sensitive to one acceleration threshold [8–10]. Once the acceleration reaches the threshold value of the sensitive component, the deformation of the sensitive component makes the electrode on it contact the fixed electrode on the substrate. For a high-g microaccelerometer, the high acceleration load can make sensitive components continue to deform significantly after they are baffled by a fixed electrode. Based on this feature, a novel high-g threshold microaccelerometer concept is developed in this article. As shown in Figure 1(a), the sensitive component is a beam-mass structure. On the beam’s bottom there are some disconnected electrodes, as shown in Figure 1(b), and these electrodes are named movable electrodes. Under each movable electrode, there are a couple of fixed electrodes on the substrate with an initial gap to the movable electrode, and meanwhile these fixed electrodes play roles as stoppers. The two fixed electrodes of each couple are disconnected in normal status as shown in Figure 1(c), and they are connected by the movable electrode when the movable electrode contacts them. Different from traditional threshold microaccelerometers, the sensitive component continues to be sensitive to further acceleration increases when the contact has happened, which can make one or some of the remainder of uncontacted fixed electrode-couples come into contact state and also can make one or more contacted fixed electrode-couples return to noncontact state. Consequently one sensitive component can be used to detect different acceleration thresholds. This design greatly reduces the sensor’s size. It should be mentioned that the structure in Figure 1 is only one illustration of a multiple-contact mode structure, and in fact different variations of the structure are possible.

**Figure 2.**
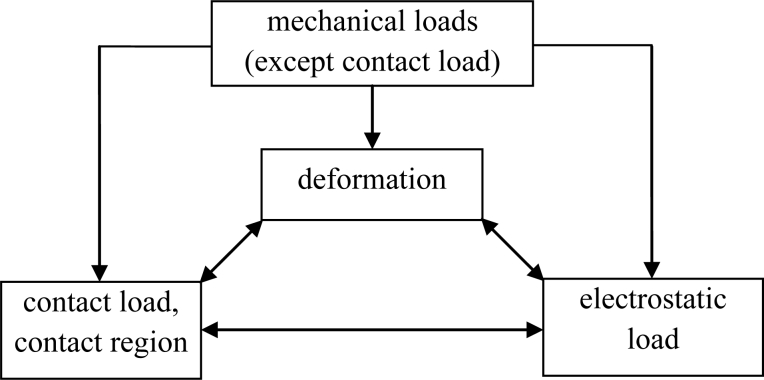
Coupling relationship.

The modeling is complex. The deformation interacts with contact load and contact region, and consequently there exists a complicated coupling relationship between contact region, contact load, other mechanical loads, electrostatic load and deformation, as shown in Figure 2 where the bidirectional arrow indicates a two-way interaction relationship and the single directional arrow indicates a one-way relationship with the action direction along the arrow.

Compared with traditional contact mode, multiple-contact mode is more complex because the contact region is discrete and stoppers’ heights are non-uniform. However, prior models of multiple-contact RF switches aren’t universal, because the multiple-contact is only in a zipper-like way. In this special multiple-contact mode, fixed stoppers contact a movable cantilever sequentially according to the order of stoppers’ locations along the cantilever’s length direction, and the stopper maintains contact with the cantilever once the contact has happened [1,2]. In zipper-like multiple-contact mode, the part of the beam which spans between two adjacent contacted stoppers, or the part of the beam which spans between the beam’s fixed end and the nearest contacted stopper, is thought to be independent of other parts, therefore the modeling of a multiple-contact beam is simplified to the modeling of a noncontact beam, and contact load isn’t taken into account in the modeling. To realize the zipper-like mode, stoppers’ height is restricted and usually each stopper has a same height. However, in the multiple-contact threshold microaccelerometer, stopper height is a key factor that influences the sensor’s resolution and measurement range. Furthermore, unlike electrostatically driven RF switches in which a high contact load and consequently low contact resistance [11–13] can be realized by high voltage and small electrode distances, the multiple-contact threshold microaccelerometer adjusts the contact load mainly by adjusting stopper heights and distributions. All these factors result in a complicated stopper height and distribution design, therefore the contact between the beam and stoppers isn’t in a zipper-like way, and calculations of deformation and contact load are very complex. In other words, to afford better flexibility to designers, a universal model of beam’s multiple-contact mode is needed, which will be applicable to different devices including threshold microaccelerometers, micromachined RF switches and microgrippers.

Besides the complicated coupling relationship and discrete contact region, in this article our multiple-contact analysis also takes into account the following facts: first, contact load influences beam’s deformation and is a crucial factor to ensure electrodes’ low contact resistance [11–13]. Second, with the increasing of loads, the stoppers’ contact sequence can be not in accordance with the order of their locations in the beam’s length direction. Third, the contact/noncontact state and contact load of any stopper indirectly influences other stopper’s contact/noncontact state and contact load. Fourth, a contacted stopper can shift back to a noncontact state with increasing load. This is because after a contact with the beam, the stopper affords a fulcrum to the beam and the beam can be regarded as a flexible lever, inducing a seesaw-like effect which makes one or more stoppers at one side of the fulcrum shift from contact state to noncontact state.

Analytical methods have been used in the analysis of traditional contact mode where the contact region is continuous and the movable structure is size-uniform, usually based on some simplification assumptions [14–17]. For the multiple-contact analysis, because the number, locations and sizes of stoppers are arbitrary, it’s difficult to get an analytic deflection expression which comprises a number of parameters, and furthermore the difficulty is aggravated if the movable structure’s shape is nonuniform. Obviously FEM (finite element method) is a choice as a numerical method for solving the problem at hand, but it’s time consuming, especially for contact analysis because contact load and contact region interact with the deformation and other loads [16], especially for any design which needs a performance comparison under different parameters. We have proposed an electromechanical coupling analysis method which is advisable for noncontact structures with stepped, curved or uniform shapes, under concentrated or distributed loads [18]. In this article, this method is developed further to afford a universal tool for multiple-contact analysis, and based on it, a design example of a high-g threshold microaccelerometer is proposed.

## 2. Modeling

### 2.1. Beam’s Deflection Expression

As the movable electrode obtained by microfabrication process is very thin, usually under 0.1 μm, its height is negligible for the modeling. As the gap between the two electrodes of each fixed electrode-couple is very small and beam’s stiffness in the width direction is very high, the two electrodes are regarded as one stopper.

**Figure 3.**
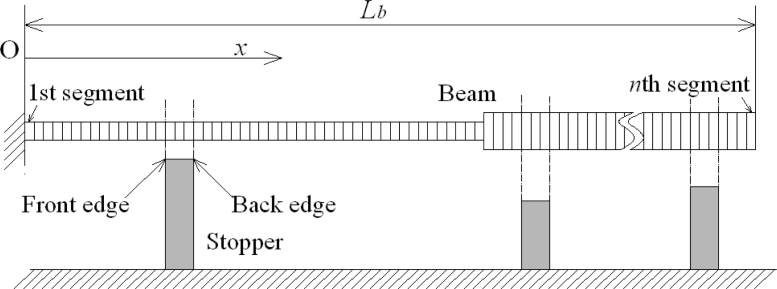
Segment division of beam.

Take the cantilever for example. As shown in Figure 3, the beam with a length *L_b_* is divided into *n* segments along its length direction. The beam can have a curved or stepped shape, and the segment division should meet the demand that beam’s width steps and height steps all are located at some segments’ edges. It’s also demanded that in the beam’s length direction either the front edge or back edge of each stopper has a location that is the same as that of the edge of some segment of the beam. From the cantilever’s fixed end to its free end, the cantilever’s segments are marked as the 1st, 2nd, … *i*th, …*n*th segment. The coordinate value in cantilever’s length direction is recorded as *x* and the coordinate origin is located at cantilever’s fixed end. *L_i-1_* ≤ *x* ≤ *L_i_* at cantilever’s *i*th segment, *L*_0_ = 0 and *L_n_* = *L_b_*. The contact load is discretised into concentrated loads applied at *x* = *L_i_* (*i* = 1,2…*n*). The resultant of the contact load applied at cantilever’s region from *x* = 0 to *x* = *L_i_* is recorded as *F_i_* (*i* = 1,2…*n*) and it’s assumed that *F*_0_ = 0. Then the resultant contact load for the whole cantilever is *F_n_*, and the contact load of the *i*th segment is *F_i_* – *F_i_*_−1_.

Based on material or plate mechanics [19,20], the following differential equation of a beam’s deflection exists:

(1) $$D_{i}\;\frac{d^{3}w_{i}(x)}{dx^{3}}\;=\;-Q_{i}(x)\;(L_{i-1}\;\leq \;x\;\leq \;L_{i})$$

where *w_i_* is the deflection of beam’s *i*th segment, *Q_i_* is the vertical shearing load per unit width at beam’s *i*th segment. *D_i_* is the flexural rigidity of beam’s *i*th segment:

(2) $$D_{i}\;=\;\frac{Eh_{i}^{3}}{12}$$

where *E* is Young’s modulus. *h_i_* is the height of beam’s *i*th segment.

Assume *q_i_* as the distributed area load applied at beam’s *i*th segment but not including the contact load which has been discretised into concentrated loads, *i.e.*,:

(3) $$q_{i}\;=\;\rho h_{i}a\;+\;\frac{{ɛ}_{0}U^{2}}{2{\left[d_{i}\;-\;(1\;-\;1/{ɛ}_{r})h_{\text{die}}\;-\;w_{i}\right]}^{2}}$$

where *ρ* is the beam’s density. *a* is acceleration. *ɛ*_0_ is vacuum permittivity. *U* is the voltage applied between the movable electrode and the fixed electrode. If a dielectric layer is located between the movable electrode and the fixed electrode, its height and relative permittivity are assumed as *h_die_* and *ɛ_r_* respectively. The term *d*_i_ is the initial distance between the movable electrode at beam’s *i*th segment and the fixed electrode. During the calculation of *q_i_*, the deflection *w_i_* inside beam’s *i*th segment is assumed to be uniform. The resultant of *q_i_* (*i* = 1,2…*n*) at the whole beam is recorded as *S_q_*, and:

(4) $$S_{q}\;=\;\sum_{i=1}^{n}q_{i}b_{i}\;(L_{i}\;-\;L_{i-1})$$

where *b_i_* is the width of beam’s *i*th segment. The reaction load at the cantilever’s fixed end is *S_q_* + *F_n_*. There exist:

(5) $$Q_{i}\;(x)\;=\;\frac{S_{q}\;+\;F_{n}\;-\;\sum_{k=1}^{i-1}q_{k}b_{k}\;(L_{k}\;-\;L_{k-1})\;-\;q_{i}b_{i}\;(x\;-\;L_{i-1})\;-\;F_{i-1}}{b_{i}}\;=\;G_{i}\;+\;\frac{F_{n}\;-\;F_{i-1}}{b_{i}}\;-\;q_{i}x\;(L_{i-1}\;<\;x\;\leq \;L_{i})$$

(6) $$G_{i}\;=\;\frac{S_{q}\;-\;\sum_{k=1}^{i-1}q_{k}b_{k}\;(L_{k}\;-\;L_{k-1})}{b_{i}}\;+\;q_{i}L_{i-1}$$

*G_i_* is introduced to decrease calculation amount and make expressions concise. When the beam has a uniform width and is subjected to a uniformly distributed area load *q*, *G_i_* remains constant to become *qL_b_*. For example, when only an acceleration load is applied to an equal-height and equal-width cantilever, *G_i_* is constant and equal to *ρh_b_L_b_a*, where *h_b_* is beam’s height.

Substituting (5) into (1) and solving the resulting equation, we obtain the following expression for the cantilever’s deflection:

(7) $$w_{i}\;(x)\;=\;A_{i}x^{2}\;+\;B_{i}x\;+\;C_{i}\;+\;\frac{F_{i-1}\;-\;F_{n}}{6b_{i}D_{i}}x^{3}\;-\;\frac{G_{i}}{6D_{i}}x^{3}\;+\;\frac{q_{i}}{24D_{i}}x^{4}\;(L_{i-1}\;\leq \;x\;\leq \;L_{i})$$

In Equation (7)*F_i_*_−1_ and *F_n_* can be regarded as undetermined parameters similar to *A_i_*, *B_i_*, *C_i_*. When *F_i_* = 0 (*i* = 0,1…*n*), Equation (7) degenerates to the deflection expression under noncontact mode whose high accuracy has been verified by our prior studies [18].

The cantilever’s slope is:

(8) $$\theta_{i}\;(x)\;=\;\frac{dw_{i}\;(x)}{dx}\;=\;2A_{i}x\;+\;B_{i}\;+\;\frac{F_{i-1}\;-\;F_{n}}{2b_{i}D_{i}}x^{2}\;-\;\frac{G_{i}}{2D_{i}}x^{2}\;+\;\frac{q_{i}}{6D_{i}}x^{3}\;(L_{i-1}\;\leq \;x\;\leq \;L_{i})$$

and the cantilever’s moment is:

(9) $$M_{i}\;(x)\;=\;b_{i}D_{i}\;\frac{dw_{i}^{2}\;(x)\;}{dx}\;=\;2A_{i}b_{i}D_{i}\;+\;(F_{i-1}\;-\;F_{n})x\;-\;G_{i}b_{i}x\;+\;\frac{q_{i}b_{i}}{2}x^{2}\;(L_{i-1}\;\leq \;x\;\leq \;L_{i})$$

### 2.2. Condition Equations

#### 2.2.1. Continuity Condition Equations

The *i*th segment and the (*i* + 1)th segment (*i* = 1,2…*n* – 1) have the same deflections, slopes and moments at their common edge *x* = *L_i_*, therefore there exist the following equations:

(10) $$\begin{array}{l} L_{i}^{2}A_{i}\;+\;L_{i}B_{i}\;+\;C_{i}\;-\;L_{i}^{2}A_{i+1}\;-\;L_{i}B_{i+1}\;-\;C_{i+1}\;+\;\frac{L_{i}^{3}}{6b_{i}D_{i}}F_{i-1}\;-\;\frac{L_{i}^{3}}{6b_{i+1}D_{i+1}}F_{i}\;-\;\frac{L_{i}^{3}}{6}\left(\frac{1}{b_{i}D_{i}}-\frac{1}{b_{i+1}D_{i+1}}\right)F_{n}\\ =\frac{L_{i}^{3}}{6}\left(\frac{G_{i}}{D_{i}}\;-\;\frac{G_{i+1}}{D_{i+1}}\right)\;-\;\frac{L_{i}^{4}}{24}\left(\frac{q_{i}}{D_{i}}\;-\;\frac{q_{i+1}}{D_{i+1}}\right) \end{array}$$

(11) $$\begin{array}{l} 2L_{i}A_{i}\;+\;B_{i}\;-\;2L_{i}A_{i+1}\;-\;B_{i+1}\;+\;\frac{L_{i}^{2}}{2b_{i}D_{i}}\;F_{i-1}\;-\;\frac{L_{i}^{2}}{2b_{i+1}D_{i+1}}F_{i}\;-\;\frac{L_{i}^{2}}{2}\left(\frac{1}{b_{i}D_{i}}\;-\;\frac{1}{b_{i+1}D_{i+1}}\right)F_{n}\\ =\;\frac{L_{i}^{2}}{2}\;\left(\frac{G_{i}}{D_{i}}\;-\;\frac{G_{i+1}}{D_{i+1}}\right)\;-\;\frac{L_{i}^{3}}{6}\left(\frac{q_{i}}{D_{i}}\;-\;\frac{q_{i+1}}{D_{i+1}}\right) \end{array}$$

(12) $$2b_{i}D_{i}A_{i}\;-\;2b_{i+1}D_{i+1}A_{i+1}\;+\;L_{i}F_{i-1}\;-\;L_{i}F_{i}\;=\;L_{i}(G_{i}b_{i}\;-\;G_{i+1}b_{i+1})\;-\;\frac{L_{i}^{2}}{2}(q_{i}b_{i}\;-\;q_{i+1}b_{i+1})$$

#### 2.2.2. Boundary Condition Equations

At a cantilever’s fixed end, the deflection and slope both are zero, therefore:

(13) $$C_{1}\;=\;0$$

(14) $$B_{1}\;=\;0$$

At a cantilever’s free end, the moment is zero, therefore:

(15) $$2b_{n}D_{n}A_{n}\;+\;L_{n}F_{n-1}\;-\;L_{n}F_{n}\;=\;G_{n}b_{n}L_{n}\;-\;q_{n}b_{n}L_{n}^{2}/2$$

If a cantilever’s *i*th segment is contacted, its deflection equals to its initial gap to the stopper. Recording the initial gap as *g_i_*, we may write:

(16) $$L_{i}^{2}A_{i}\;+\;L_{i}B_{i}\;+\;C_{i}\;+\;\frac{L_{i}^{3}}{6b_{i}D_{i}}F_{i-1}\;-\;\frac{L_{i}^{3}}{6b_{i}D_{i}}F_{n}\;=\;g_{i}\;+\;\frac{G_{i}}{6D_{i}}L_{i}^{3}\;-\;\frac{q_{i}}{24D_{i}}L_{i}^{4}$$

If a cantilever’s *i*th segment is uncontacted, due to the absence of a contact load on it, we have the expression:

(17) $$F_{i}\;-\;F_{i-1}\;=\;0$$

It should be mentioned that when the fixed electrode is curved, the above equations also are applicable only by regarding *d_i_* at Equation (3) and *g_i_* at Equation (16) as functions of the *i*th segment’s location, similarly to what we have done for a noncontact cantilever [18], almost introducing no additional complexity to the calculation. If pre-stress is taken into account, the deflection expression will be different because a term which is the product of the pre-stress per unit width and deflection’s 1st order derivative should be added into the deflection differential Equation (1) [20], but the above modeling method also is applicable in that case, and according to our prior studies on the noncontact problem [18] it is known that it is not difficult to solve this deflection differential equation considering multiple-contact mode.

## 3. Algorithm

Based on Equations (10–17), a system of linear equations as the following is obtained:

(18) JX = K

Variables *A_i_*, *B_i_*, *C_i_* and *F_i_* (*i* = 1,2…*n*) form a 4*n* × 1 matrix *X. J* is a 4*n* × 4*n* sparse matrix whose nonzero elements are coefficients of *A_i_*, *B_i_*, *C_i_* and *F_i_* in Equations (10–17). When contact/noncontact regions remain unchanged, the value of *J* is independent of the deflection. When contact/noncontact regions vary, the condition equations of the new contact/noncontact segment are also changed and consequently the value of *J* is changed. In Equations (10–17), terms independent of *A_i_*, *B_i_*, *C_i_* and *F_i_*, *i.e.*, the right hand parts of Equations (10–17), constitute a 4*n* × 1 matrix *K*, and *K*’s value should be updated with updated deflections because the electrostatic load and consequently *q_i_*, *G_i_* are functions of the deflection.

Each stopper’s top, *i.e.*, the stopper’s contact surface, is replaced by several possible contact points distributed along the beam’s length direction. When all possible contact points of one stopper are contacted, it means the stopper’s top completely contacts the beam, otherwise it means the stopper’s top only partially contacts the beam. Among all possible contact points of all stoppers, any one of them, any two of them, any three of them, and so forth up to all of them form a set of possible combinations of stopper contact points. Among all these possible combinations, one of them must match the actual situation.

The beam’s deformation calculation process is as follows:

- Select one of the possible combinations of stopper contact points.
- Calculate *J*.
- Calculate *K*.
- Solve *JX* = *K.*
- Calculate the deflection by substituting values of *A_i_*, *B_i_*, *C_i_* and *F_i_* into Equation (7).
- Judge whether the deflection is convergent under the current assumption of stoppers’ contact points. If not, update the electrostatic load according to the beam’s new calculated deflection, and then repeat steps (c–f). If yes, go to step (g).
- Judge whether each uncontacted segment of the beam has a calculated deflection smaller than the segment’s initial gap to the stopper or substrate and whether each contact load is a push load but not a pull load. If not, select another possible combination of stopper contact points, and then repeat steps (b–g). If yes, the calculation ends.

The reason for the convergence judgment in step (f) is that the electrostatic load interacts with the beam’s deflection, so this step is necessary only when an electrostatic load is applied. The reason of the contact load direction judgment in step (g) is that there is no limitation on *F_i_* in Equation (7) and consequently, a virtual contact load which is a pull load may result. The judgment of the contact load’s direction only needs to check signs of *F_i_* – *F_i_*_−1_(*i* = 2,3…*n*) and *F*_1_, *i.e.*, the sign of the contact load applied at each segment.

In addition, to speed up the calculations, a dynamic meshing can be used, *i.e.*, a coarse segment division is used during the search of the correct combination of contact points, and a fine segment division is used after the correct combination of contact points has been found.

Pull-in analysis can be realized based on the above algorithm, by an iteration of deflection calculations under different voltages, similarly to what we have reported for a noncontact beam [18]. Different types of stresses can be obtained because they are functions about deflection.

## 4. Validation

The novel contact analysis method was validated by FEM, using CoventorWare, which is a widely employed CAD software suite for MEMS that can realize contact analysis combined with electromechanical coupling analysis. To verify the analysis method’s applicability to different load types and different devices, both electrostatic load and mechanical load were applied in the validation. Accordingly, in this section, different to Figure 1, the movable electrode completely covers the cantilever’s bottom, and stoppers are all regarded to be insulative with a relative permittivity of 9. Furthermore, a fixed electrode located on the substrate is assumed to be under the beam, and its length and width are not smaller than those of the cantilever. In the validation, there are five stoppers. The cantilever has three parts with different dimensions, and starting from the fixed end, they are identified as the 1st, 2nd and 3rd parts respectively. Structure parameters in the validation are listed in Table 1. The cantilever’s density, Young’s modulus and Poisson’s ratio are 2,500 kg/m^3^, 169 Gpa and 0.3, respectively. Acceleration *a* (unit: g = 9.8 m/s^2^) is applied to the cantilever, and voltage *U* is applied between the movable electrode and the fixed electrode. The validation results are shown in Table 2. The cantilever’s average deflection resulting from the novel model and CoventorWare are recorded as *w̄* and *w̄*^′^, respectively. The contact loads applied by the 1st, 2nd, 3rd, 4th and 5th stoppers are recorded as *F*_1_, *F*_2_, *F*_3_, *F*_4_ and *F*_5_, respectively, when they result from the above model, and as *F*_1_^′^, *F*_2_^′^, *F*_3_^′^, *F*_4_^′^ and *F*_5_^′^, respectively, when they are produced by CoventorWare. A zero contact load means no contact. Acceleration, voltage and stoppers’ initial distance to the cantilever are changed in Table 2. The maximum absolute value of relative error of average deflection is 0.34%, and the maximum absolute value of relative error of contact load is 5.16%. Additionally, cases 1, 2 or cases 4, 5 in Table 2 illustrate that as the loads increase, the stoppers’ contact sequence maybe isn’t in accordance with the order of stoppers’ locations along the beam’s length direction.

**Figure 4.**
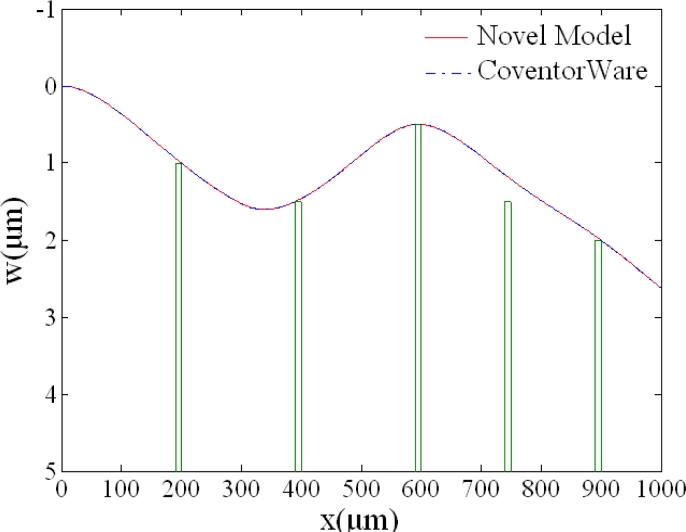
Deflection curves of case 4 in Table 2.

The novel model’s accuracy is illustrated in Figure 4. The green solid lines are stoppers’ initial gaps to the cantilever. Deflection curves of case 4 in Table 2 resulted from the novel model and CoventorWare, respectively, and it can be found that the two curves almost overlap.

3,000∼4,000 elements in CoventorWare make the calculation result trend to be stable, regardless of any further increase of the number of segments, and each case of Table 2 requires 1–3 hours to execute in CoventorWare. When 100 coarse segments division and 500 fine segments division of the beam, and two contact points of each stopper are used, the calculation result of the novel model has a negligible difference compared with the calculation result from CoventorWare. Furthermore the novel model only took 2.7 s to 9.6 s to calculate each case though the model is realized in Matlab scripting language, under a same hardware environment: a 2.67 GHz CPU, 4 GB physical memory, 3 GB virtual memory. If each case’s complex modeling process in CoventorWare is taken into account, or if the novel model is realized by C language or other programming languages, the novel model’s speed advantage will be more significant.

## 5. A Design Example of Multiple-Contact High-g Threshold Microaccelerometer

Multiple-contact beam’s complex deformation and the reliability demand on contact load make the design of the novel microaccelerometer need a search for suitable structure parameters values, resulting in a large amount of calculations. The above calculation model’s high accuracy and high speed make it competent to realize the design. One design example is shown in the following.

In the current design, the beam-mass structure as shown in Figure 1 is demanded to detect five acceleration thresholds: 1,000 g, 2,000 g, 3,000 g, 4,000 g and 5,000 g. Another design target is to meet the reliability demand on contact load. Based on a series of prior studies, Oberhammer and Stemme have summarized that gold affords a stable and low enough contact resistance to micromachined switches when the contact load is above 50∼100 μN [11], and Ma’s study on micromachined RF switches has shown that gold’s contact resistance is stable and low even when contact load is under 30 μN [12]. Usually the operation cycle number of threshold microaccelerometers is much smaller than that of RF switches, which makes threshold microaccelerometers have a relatively lower contact load demand [13]. Therefore in the current design it’s required that the contact load of each fixed electrode-couple isn’t lower than 100 μN, assuming that electrodes are made of gold.

Structure parameters of the designed case are listed in Table 3. There are five fixed electrode-couples. The fixed electrode needn’t to be long in the beam’s length direction, and in fact long fixed electrode will decrease the sensitivity because beam’s deformation becomes relatively difficult after the contact.

The beam-mass structure’s density, Young’s modulus and Poisson’s ratio are 2,500 kg/m^3^, 169 Gpa and 0.3, respectively. Each fixed electrode-couple’s top area is small, and only a low voltage is needed, so electrostatic load is negligible compared with the high acceleration load, which results in a higher calculation speed because no convergence judgment is needed. The whole of the beam-mass structure is regarded as a cantilever with height step and width step, affording high calculation accuracy. The 1st, 2nd, 3rd, 4th and 5th fixed electrode-couple’s contact loads *F*_1_, *F*_2_, *F*_3_, *F*_4_ and *F*_5_ resulting from the novel model are listed in Table 4, and the design is verified by CoventorWare. In the table, zero contact load means no contact happens between the movable electrode and the fixed electrode-couple, *i.e.*, the two electrodes in the fixed electrode-couple aren’t connected. Under different threshold accelerations, there are different combinations of connected/disconnected fixed electrode-couples. There are always two fixed electrode-couples that are in connect state simultaneously when under the 2nd to 5th threshold accelerations. Therefore when one fixed electrode-couple shifts from connect state to disconnect state due to the seesaw-like effect, another electrode-couple has been in connect state, preventing the phenomenon that more than one acceleration levels inducing all fixed electrode-couples being in disconnect state. An array of such multiple-contact beam-mass component will compose a threshold microaccelerometer with a wide measurement range but of a smaller size compared to traditional threshold microaccelerometers.

## 6. Conclusions

A novel analysis method and a novel calculation model are developed for the analysis of beams’ multiple-contact mode. Deflection, contact load and contact region are obtained directly, with subsequent validation by CoventorWare. The contact analysis includes an electromechanical coupling analysis, and consequently pull-in voltage calculation and so on also are realizable. Though the analysis method is accurate, it isn’t complicated, and consequently it’s time-saving and has a good repeatability.

A novel design of a high-g threshold microaccelerometer is developed, characterized by the advantage that each sensitive component works under multiple-contact mode with multiple acceleration thresholds. This design reduces the sensor’s size considerably. In the design, low contact resistance is ensured by making the contact load above a demanded value.

As a universal model for beams’ contact mode, the model developed in this article can be degenerated to calculate the deflection and contact load at traditional low-g threshold microaccelerometers, microswitches, microgrippers and so on. The novel model also is applicable for acceleration threshold adjustments and built-in self-tests in low-g threshold microaccelerometers where an electrostatic load comparable to the acceleration load is applied by a fixed electrode besides the beam or mass [8–10]. Furthermore, the model can be degenerated to analyze the zipper-like multiple-contact mode which has been used in RF switches.

## Figures and Tables

**Table 1.** Structure parameters for the validation.

**Structure parameter**	**Value (μm)**
Cantilever’s total length	1,000
Length of cantilever’s 1st part	300
Width of cantilever’s 1st part	20
Height of cantilever’s 1st part	20
Length of cantilever’s 2nd part	500
Width of cantilever’s 2nd part	10
Height of cantilever’s 2nd part	15
Length of cantilever’s 3rd part	200
Width of cantilever’s 3rd part	20
Height of cantilever’s 3rd part	20
Length of each stopper	10
1st stopper’s distance to cantilever’s fixed end in the length direction	190
1st stopper’s initial gap to the cantilever	*g*_1_
2nd stopper’s distance to cantilever’s fixed end in the length direction	390
2nd stopper’s initial gap to the cantilever	*g*_2_
3rd stopper’s distance to cantilever’s fixed end in the length direction	590
3rd stopper’s initial gap to the cantilever	*g*_3_
4th stopper’s distance to cantilever’s fixed end in the length direction	740
4th stopper’s initial gap to the cantilever	*g*_4_
5th stopper’s distance to cantilever’s fixed end in the length direction	890
5th stopper’s initial gap to the cantilever	*g*_4_
Fixed electrode’s initial gap to the cantilever	5

**Table 2.** Model’s Validation.

		**Case 1**	**Case 2**	**Case 3**	**Case 4**	**Case 5**
Initial distance between stopper and cantilever	*g_1_*(μm)	0.5	0.5	0.5	1	1
	*g_2_*(μm)	0.5	0.5	0.5	1.5	1.5
	*g_3_*(μm)	0.5	0.5	0.5	0.5	0.5
	*g_4_*(μm)	1	1	0.5	1.5	1.5
	*g_5_*(μm)	2	2	2	2	2
Applied load	*a*(g)	5 × 10^5^	1 × 10^6^	5 × 10^5^	1 × 10^6^	0
	*U*(V)	50	100	50	100	100
	*w̄* (μm)	0.79950	0.82300	0.70994	1.1417	0.64497
Deflection	*w̄*^′^ (μm)	0.80079	0.82419	0.71238	1.1426	0.64468
	(*w̄ – w̄*^′^) / *w̄*^′^	−0.16%	−0.14%	−0.34%	−0.08%	0.04%
	*F*_1_ (μN)	0	612.8	0	529.3	0
	*F*_1_^′^ (μN)	0	646.2	0	557.9	0
	(*F*_1_ – *F*_1_^′^) / *F*_1_^′^	/	−5.16%	/	−5.13%	/
Calculated contact load (Validation value of CoventorWare is signed with superscript apostrophe.)	*F*_2_ (μN)	624.8	1,201.4	679.2	499.7	0
	*F*_2_^′^ (μN)	625.1	1,187.1	680.8	500.7	0
	(*F*_2_ – *F*_2_^′^) / *F*_2_^′^	−0.05%	1.20%	−0.24%	−0.20%	/
	*F*_3_ (μN)	349.5	665.4	0	1,493.8	54.2
	*F*_3_^′^ (μN)	349.4	669.6	0	1,486.2	55.4
	(*F*_3_ – *F*_3_^′^) / *F*_3_^′^	0.03%	−0.63%	/	0.51%	−2.17%
	*F*_4_ (μN)	340.8	560.0	851.0	0	0
	*F*_4_^′^ (μN)	339.1	557.3	846.0	0	0
	(*F*_4_ – *F*_4_^′^) / *F*_4_^′^	0.50%	0.48%	0.59%	/	/
	*F*_5_ (μN)	904.3	1,950.7	687.0	2,118.0	64.3
	*F*_5_^′^ μN)	904.9	1,950.2	690.1	2,119.2	66.2
	(*F*_5_ – *F*_5_^′^) / *F*_5_^′^	−0.07%	0.03%	−0.45%	−0.06%	−2.87%

**Table 3.** Structure parameters of the design example.

**Structure parameter**	**Value (μm)**
Beam’s length	800
Beam’s width	20
Beam’s height	20
Mass’ length	200
Mass’ width	50
Mass’ height	180
Length of each fixed electrode-couple	10
1st fixed electrode-couple’s distance to beam’s fixed end in the length direction	240
1st fixed electrode-couple’s initial gap to the beam	0.3
2nd fixed electrode-couple’s distance to beam’s fixed end in the length direction	315
2nd fixed electrode-couple’s initial gap to the beam	0.6
3rd fixed electrode-couple’s distance to beam’s fixed end in the length direction	365
3rd fixed electrode-couple’s initial gap to the beam	0.9
4th fixed electrode-couple’s distance to beam’s fixed end in the length direction	415
4th fixed electrode-couple’s initial gap to the beam	1.3
5th fixed electrode-couple’s distance to beam’s fixed end in the length direction	455
5th fixed electrode-couple’s initial gap to the beam	1.7

**Table 4.** Contact load of the design example (validation value from CoventorWare is in brackets).

	**case 1**	**case 2**	**case 3**	**case 4**	**case 5**
*a*(g)	1,000	2,000	3,000	4,000	5,000
*F*_1_ (μN)	101.5 (100.9)	117.7 (113.4)	0	0	0
*F*_2_ (μN)	0	148.3 (150.7)	128.3 (124.7)	0	0
*F*_3_ (μN)	0	0	219.2 (221.7)	167.9 (164.2)	0
*F*_4_ (μN)	0	0	0	249.9 (252.7)	198.7 (194.8)
*F*_5_ (μN)	0	0	0	0	276.3 (279.5)
